# 656. Sulbactam-Durlobactam MIC Determination: Comparative Evaluation of the New ETEST^®^ SUD to the CLSI 2021 Broth Microdilution Method

**DOI:** 10.1093/ofid/ofab466.853

**Published:** 2021-12-04

**Authors:** Veronique Sauvonnet, Elodie Escoffier, Christine Franceschi, Diane Halimi, Roland Martelin, Laetitia Pellet, Gilles Zambardi

**Affiliations:** Biomerieux SA, La Balme Les Grottes, Rhone-Alpes, France

## Abstract

**Background:**

Species belonging to the *Acinetobacter baumannii-calcoaceticus* (ABC) complex, such as *A. baumannii, A. pittii* and *A. nosocomialis,* are a major cause of hospital acquired infections and outbreaks with increasing occurrence of multidrug-resistance. Sulbactam-durlobactam (SUD), a combination of one active β-lactam antibiotic (sulbactam) with a new β-lactamase inhibitor (durlobactam), is currently being tested in a phase 3 clinical trial by Entasis Therapeutics for the treatment of serious infections caused by ABC, including multidrug-resistant strains. At the same time, an ETEST^®^ SUD (sulbactam-durlobactam - MIC range 0.004/4-64/4 µg/mL) has been developed and calibrated versus the broth microdilution reference method (BMD) as described by the Clinical and Laboratory Standards Institute (CLSI). This test is intended to determine the MIC of sulbactam-durlobactam for species of the ABC complex. The aim of this study was to perform a first comparative study of ETEST SUD with the CLSI BMD method on a panel of 263 isolates.

**Methods:**

The panel consisted of 204 *A. baumannii*, 29 *A. pittii,* 30 *A. nosocomialis*, including 24 SUD-resistant strains, and one CLSI QC strain. BMD was performed using the 2021 CLSI guidelines. ETEST SUD was evaluated using the standard ETEST procedure for *Acinetobacter* spp. (inoculum 0.5 McFarland, Mueller Hinton medium, incubation at 35°C for 20-24h). For each method, the MIC was read at complete inhibition of visible growth. To determine category agreement (CA) and error rates, the sulbactam-durlobactam provisional breakpoint of 4 µg/mL was applied.

**Results:**

The QC strain MICs were in the expected range with reproducible results. The essential MIC agreement [EA, ±1 dilution] was 97.7% without any tendency to over- or underestimate the MIC when compared to BMD. The CA was 98.5%. Two Very Major Errors, both within the EA, and two Major Errors, one within the EA, were observed.

**Conclusion:**

In this study, the ETEST SUD was found to be equivalent to the CLSI reference method. MIC end points were easy to read. With a 15-dilution range and simplicity of use, ETEST SUD could represent a valuable tool for MIC determination and could be an alternative to BMD.

For Research Use Only. The performance characteristics of this product have not been established yet.

**Disclosures:**

**All Authors**: No reported disclosures

